# From the Performance to the Essence: The Biological Mechanisms of How Tantalum Contributes to Osteogenesis

**DOI:** 10.1155/2020/5162524

**Published:** 2020-07-27

**Authors:** Hu Qian, Ting Lei, Zhimin Ye, Yihe Hu, Pengfei Lei

**Affiliations:** ^1^Department of Orthopedics, Xiangya Hospital of Central South University, 87 Xiangya Road, Changsha, Hunan 410008, China; ^2^Xiangya School of Medicine, Central South University, 172 Tongzipo Road, Changsha, 410008 Hunan, China

## Abstract

Despite the brilliant bioactive performance of tantalum as an orthopedic biomaterial verified through laboratory researches and clinical practice in the past decades, scarce evidences about the essential mechanisms of how tantalum contributes to osteogenesis were systematically discussed. Up to now, a few studies have uncovered preliminarily the biological mechanism of tantalum in osteogenic differentiation and osteogenesis; it is of great necessity to map out the panorama through which tantalum contributes to new bone formation. This minireview summarized current advances to demonstrate the probable signaling pathways and underlying molecular cascades through which tantalum orchestrates osteogenesis, which mainly contain Wnt/*β*-catenin signaling pathway, BMP signaling pathway, TGF-*β* signaling pathway, and integrin signaling pathway. Limits of subsistent studies and further work are also discussed, providing a novel vision for the study and application of tantalum.

## 1. Introduction

Tantalum (Ta), a refractory metal, was well-known for its excellent biocompatibility, corrosion resistance, and bioactivity, making Ta a desirable biomaterial for medical applications [1–4]. Since the introduction of trabecular metal (TM), a novel porous Ta implant for acetabular cups, by Zimmer (Warsaw, IN, USA) in the early 21st century, porous tantalum implants have been widely applied in bone and joint reconstruction surgery, with more than 800,000 TM used as early as in 2012 [5–7].

Despite these inspiring advantages of Ta, orthopaedic implants made of titanium (Ti) and its alloys are still the prominent choice for orthopedists [8, 9]. As such, many in vitro, in vivo, and clinical studies were carried out to compare the biological performance of Ta and Ti, synergistically demonstrating the better osteogenic property of tantalum with higher expression of osteogenic indicators in Ta group [10–12]. These osteogenic indicators included alkaline phosphatase (ALP), cellular mineralization, type I collagen (COL I), osteocalcin (OCN), and osteopontin (OPN). The outstanding osteogenic performance of Ta intrigued researchers and induced them to explore the underlying mechanisms. Bone regeneration was an intricate process that involves the activation or inhibition of multiple signaling pathways rather than simple reflection [13, 14]. In recent years, it has been revealed that Ta was associated with a great deal of classical osteogenic signaling pathways, as summarized in Table 1, including the Wnt/*β*-catenin signaling pathway [15], transforming growth factor-beta (TGF-*β*) and bone morphogenic proteins (BMPs) signaling pathway [16–19], mitogen-activated protein kinases (MAPKs) signaling pathway [20, 21], and integrin signaling pathway [22, 23]. The higher expression of critical molecules of the osteogenic signaling pathways in the Ta group, as compared with the Ti group, demonstrated that Ta could exert an active effect on multiple signaling pathways related to osteogenesis [24]. However, even though great progress was made in the exploration of osteogenic mechanisms of Ta, no reviews summarizing the advances were published yet. Much further research remains to be done; therefore, it could assume great significance to map out the panorama of the complex osteogenic mechanisms of Ta. In this review, we constructed a panorama consisting of multiple osteogenic signaling pathways activated by Ta according to the published studies, pointed out the limits of existing studies and outlined the future work, providing a novel vision for the study and application of tantalum.

## 2. Osteogenesis Signaling Pathways Related to Ta

### 2.1. TGF-*β* Superfamily

TGF-*β* superfamily consists of BMPs subgroup, TGF-*β*s, activin, osteoprotegerin (OPG), receptor activator for nuclear factor-*κ* B ligand (RANKL), and receptor activator for nuclear factor-*κ* B (RANK) [33, 34]. TGF-*β* superfamily members could bind the transmembrane heteromeric receptor complex comprising two kinds of receptors (type I and type II), thereby modifying intracellular proteins and molecules, the classical small mothers against decapentaplegic (Smad) complex pathway and MAPK cascade, to regulate gene expression [35]. TGF-*β* superfamily not only plays an important role in the intramembranous and endochondral ossification during the embryonic period but also orchestrates osteogenesis and osteolysis through regulating osteoblast and osteoclast [17, 18, 36, 37].

#### 2.1.1. TGF-*β* Signaling Pathway

There are three conformations of TGF-*β*s, namely, TGF-*β*1, TGF-*β*2, and TGF-*β*3, and there are two kinds of TGF-*β* receptors, namely, type I and type II TGF-*β* receptor (T*β*RI or ALK5, T*β*RII) [38]. In the classical TGF-*β* signaling pathway, the TGF-*β*s firstly interact with TGF-*β* receptor complex comprising two T*β*RIs and two T*β*RIIs, and then, the T*β*RII phosphorylates the T*β*RI, activating the receptor-activated Smads (R- Smads), Smad2 and Smad3 successively [17]. Smad2/3 binds to the common Smad (Co-Smad), Smad4, to form a complex and move into the nucleus, and then the complex interacts with other molecules to control gene expression. TGF-*β*s can also act through the nonclassical TGF-*β* signaling pathway, also known as non-Smad-dependent pathway, in which binding of TGF-*β*s with the receptor complex results in phosphorylation of TGF-*β* activation kinase1 (TAK1), thereby activating the MAP kinase kinase 3/6 (MKK3/6)/p38 signaling pathway, or extracellular signal-regulated kinase (ERK) signaling pathway [20, 39].

**Figure 1:**
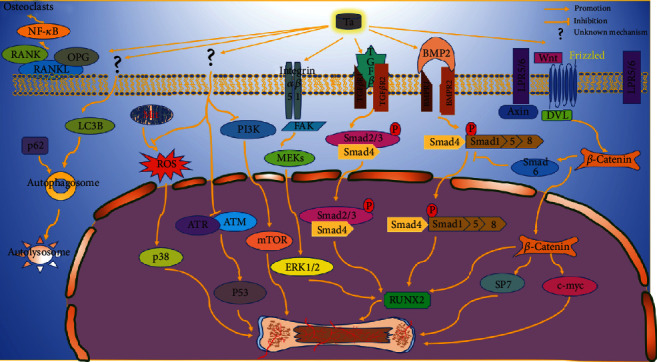
The panorama of osteogenesis signaling pathways involving Ta. OPG: osteoprotegerin; RANKL: receptor activator for nuclear factor-*κ* B Ligand; RANK: receptor activator for nuclear factor-*κ* B; FAK: focal adhesion kinase; PI3K: phosphatidylinositol 3-kinase; ERK: extracellular signal regulated kinase; Smad: small mothers against decapentaplegic; TGF-*β*: transforming growth factor-beta; BMP: bone morphogenic proteins; MAPK: mitogen-activated protein kinase; GSK3*β*: glycogen synthase kinase 3; APC: adenomatous polyposis coli; Axin: axis inhibition protein; Runx2: runt-related transcription factor 2; ATM: ataxia telangiectasia mutated; ATR: ATM-Rad3-related; LRP4/5/6: low density lipoprotein receptor-related proteins 4/5/6; DVL: disheveled; ROS: reactive oxygen species; mTOR: mammalian target of replication.

Shi et al. [16] cocultured human bone mesenchymal stem cells (hBMSCs) on Ta and titanium (Ti) disc, respectively, and observed higher expression of ALP and calcium nodules in hBMSCs cultured on Ta disc. Meanwhile, the expression of Smad3, a critical protein involved in the activation of the TGF-*β*/Smads signaling pathway, was significantly higher in the Ta group after 21 and 28 days of culturing. As shown in Figure 1, Smad2/3 triggered by Ta interacts with Smad4 and translocates into the nucleus, thereby activating osteogenic genes such as Runt-related transcription factor 2 (Runx2) to promote osteogenesis. For further verification, a pretreatment with a specific inhibitor of Smad3 (SIS3) was applied to inhibit the TGF-*β*/Smad signaling pathway. Interestingly, the productions of ALP and calcium nodules reduced gradually with the increase of SIS3, which demonstrated that Ta might stimulate osteogenesis through activating the TGF-*β*/Smad signaling pathway. Similarly, Hefni et al. implanted porous tantalum trabecular metal (PTTM) (Zimmer Biomet, USA) and Ti cylinders into bilateral mandibles of osteopenic patients [27], finding the up-regulation of TGF-*β*3 and TGF-*β*2 in the Ta group at mRNA level, as compared to the Ti group. These results indicated synergistically that Ta could promote osteogenesis through the TGF-*β* signaling pathway [40–43].

#### 2.1.2. BMP Signaling Pathway

BMPs account for the largest subgroup of the TGF-*β* superfamily, and the BMP signaling pathway is one of the most famous signaling pathways related to osteogenesis [44]. The binding of BMPs with two homomeric type II receptors activates the type I receptor [45, 46], thereby inducing the formation of the complex consisting of specific R-Smad (Smad1/5/8) and the C-Smad (Smad4). The formed complex is then translocated into the nucleus to regulate the activity of osteogenic genes [47].

Lu et al. [19] cocultured BMSCs derived from ovariectomized rats (OVX-rBMSCs) with Ta and Ti, respectively, and found better osteoinduction in the Ta group. The higher expression of Smad1, Runx2, and BMP2 in the Ta group was detected by RT-PCR and Western blot (WB), as compared with that of Ti, indicating that Ta may trigger the BMP2/Smad/Runx2 cascade [19]. As depicted in Figure 1, BMP2 activated by Ta could induce the excitation of the tetrameric receptor complex and transduced the signals to the intracellular Smad1/5/8 complex, which could get into the nucleus and combine with Smad4 and then regulates the transcription of Runx2. And in further research, both down-regulation of BMP2 and Smad1 and up-regulation of BMP2 could lead to a corresponding change of down-stream genes, Runx2 and ALP conformably, confirming the activation of the BMP signaling pathway again. It is worth noting that hBMSCs cultured on Ta exhibited enhanced osteogenesis with higher expression of Smad1 and Runx2 as compared to those cultured on other stiff materials, such as Ti, diamond-like carbon (DLC), and chromium [25]. In addition, Hefni et al. [27] implanted PTTM into mandibles of osteopenic patients to assess the osteoinductivity of Ta; up-regulation of BMP4 was observed at 2 weeks after implantation. And Bencharit et al. [10] reported better bone defects repair, as well as higher expressions of BMP 3, 4, 5, 7 in the Ta group than that of the Ti alloy group. Besides pure Ta implants, Ta coating on polyetheretherketone (PEEK) by plasma spray was also reported to enhance osteogenic properties of PEEK implants, with higher expression of BMP2 and Runx2 gene than that of the noncoated PEEK group [19, 26]. These in vitro/vivo studies demonstrated the excellent osteogenic property of Ta and revealed that Ta might stimulate the BMP signaling pathway to promote osteogenesis, especially BMP2/Smad1/Runx2 cascade.

#### 2.1.3. OPG/RANKL/RANK Signaling Pathway

The OPG/RANKL/RANK regulatory system is a coin orchestrating osteogenesis and osteolysis, and all of the three critical molecules belong to the TGF-*β* superfamily [48]. The combination and interaction between RANKL and RANK could induce osteoclastogenesis, while OPG competes with RANK to bind RANKL, thereby inhibiting the formation of osteoclasts and bone resorption [49]. As such, a low ratio of RANKL/OPG is generally regarded as the mark of osteogenesis [50, 51]. Lu et al. [19] found that OVX-rBMSCs seeded on the Ta sheets exhibited lower expression of RANKL and higher expression of OPG than that of the Ti group at mRNA level, indicating the osteogenesis potential of Ta through OPG/RANKL/RANK signaling pathway. And Shi et al. found that *μ*m-scale tantalum powder could inhibit preosteoclast cell proliferation and differentiation remarkably, as compared with Ti powder [16]. These studies revealed that Ta has the potential to inhibit osteoclastogenesis and promote osteogenesis.

### 2.2. Wnt/*β*-Catenin Pathway

Wnt/*β*-catenin pathway is involved in body development and growth, especially bone metabolism [15, 52, 53]. Under normal conditions, the *β*-catenin connects with glycogen synthase kinase 3*β* (GSK3*β*), adenomatous polyposis coli (APC), and axis inhibition protein (Axin) to form a degradative complex, which is degraded through the targeting effect of Axin to the proteasome. When the Wnt ligands bind the transmembrane receptors, low-density lipoprotein receptor-related proteins 4/5/6 (LRP4/5/6) and Frizzled, the sensitized LRP4/5/6 recruits Axin derived from the degradative complex. The absence of Axin results in the disassembly of the degradative complex under the influence of disheveled (DVL) and gives rise to the release and accumulation of *β*-catenin in the cytoplasm. Accumulative *β*-catenin gets into the nucleus and regulates transcription of osteogenesis relevant genes [15].

Shi et al. [16] found that hBMSCs seeded on Ta discs expressed a higher level of *β*-catenin, as compared to Ti discs with the same surface topography. Meanwhile, they also found a higher expression of downstream genes of the Wnt/*β* signaling pathway in the Ta group, including secreted phosphoprotein 1 (Spp1), alkaline phosphatase, liver/bone/kidney (ALPL), Runx2, Axin2, and C-myc. However, it deserves consideration that the upregulation was also observed in the expression of Smad6 which was reported to compete with Smad1/5/8 to suppress the formation of Smad4-Smad1/5/8 complex [21, 54]. Additionally, the change of genes expression of the osteoblast-like cell line (MG63) in response to *μ*m-scale tantalum powder was evaluated through DNA microarrays, and the result suggested that Ta enhanced the expression of Wnt1 signaling pathway protein 3 (WISP3) which plays an important positive role in Wnt/*β*-catenin signaling pathway [28]. These studies synergistically demonstrated that Ta may induce osteogenesis by triggering the Wnt/*β*-catenin signaling pathway [16].

### 2.3. Integrin Signaling Pathway

Integrin, a transmembrane adhesion protein, consists of *α*/*β* heterodimer and is responsible for mediating the cell-matrix and cell-cell interaction [23]. The binding of integrin and its ligands leads to the phosphorylation of integrin and recruitment of focal adhesion kinase (FAK) in the cytoplasm. Then, the FAKs activate the phosphatidylinositol 3-kinase (PI3K) or ERK1/2 to regulate the downstream genes [55]. Integrin is also implicated with osteogenesis; moreover, several integrins, such as integrin *α*v/*β*1, *α*2*β*1, and *α*5*β*1, have been reported to play a critical role in osteogenesis [23, 56–59].

As such, some researchers have tried to explore whether Ta could promote osteogenesis through the integrin signaling pathway. Lu et al. [22] found that mirror polished Ta enhanced the expression of integrin *α*5, integrin *β*1, ERK1/2, and Runx2 of rBMSCs at both protein and mRNA level, as compared with that of Ti. And they also found that inhibition of integrin *α*5, *β*1, and ERK 1/2 by siRNA resulted in down-regulation of downstream genes and impaired osteogenesis; overexpression of integrin *α*5 and *β*1 resulting from coding sequence could promote osteogenesis, and this effect could be subsequently counteracted by inhibition of ERK1/2. Analogously, Ta coating on Ti was reported to be able to enhance the expression of FAK which plays an important role in the integrin signaling pathway [29]. Increased expressions of integrin receptors, such as ITGA1, ITGA2, and ITGFGB1 were also observed in PTTM explanted from mandible of osteoporotic patients at 2 and 4 weeks after implantation [27]. All of these studies implied that Ta may promote osteogenesis through the integrin/ERK1/2/Runx2 pathway, as delineated in Figure 1.

### 2.4. Others

Besides the above-mentioned signaling pathways, Ta was reported to promote osteogenic differentiation and osteogenesis via some other mechanisms, such as the MAPK signaling pathway mediated by oxidative stress [28, 30], activation of the angiogenic specific gene [10], and even autophagy relevant mechanism [32].

Reactive oxygen species (ROS), also known as free radicals, are small molecules derived from oxygen and are involved with various biological processes, for instance, the differentiation of BMSCs [60, 61]. Wang et al. [30] found that primary osteoblasts of diabetic rabbits cultured on Ta-coated Ti exhibited better osteogenesis performance and a lower expression of ROS and p38, in comparison to pure Ti. With the addition of ROS inhibitor (NAC) or p38 inhibitor (SB203580), it was further confirmed that this difference was attributed to excessive ROS derived from mitochondrial and resultant overactivation of p38 in the Ti group, suggesting that Ta enhanced osteogenesis through inhibiting the overexpression of ROS-mediated MAPK pathway. Similarly, Lu et al. [19] also observed a lower level of ROS from OVX-rBMSCs cultured on Ta than that of the Ti group. Sollazzo et al. [28] reported that MAP3K2, a molecule in the upstream of the MAPK pathway, was up-regulated in MG63 cocultured with tantalum powder. These findings indicated that Ta may promote osteogenesis through the oxidative stress-mediated MAPK pathway, as depicted in Figure 1.

Osteogenesis was coupled with the angiogenesis of a specific vessel subtype (CD31^hi^Emcn^hi^) [62–64]. When implanted into the human mandible, PTTM enhanced significantly the expressions of specific genes accounting for angiogenesis, as compared to titanium alloy, implying that Ta may initiate the coupling process of osteogenesis and angiogenesis [3]. It was interesting that the Ta nanoparticles (Ta-NPs) could be phagocytized by osteoblasts and improve the proliferation ability of osteoblasts [32]. And the whole-genome expression analysis (WGEA) of hBMSCs, cultured on ultrasmooth Ta and Ti, showed that Ta deregulated the expression of upstream genes of p53 signaling pathway, including ataxia telangiectasia mutated (ATM) and ATM-Rad3-Related (ATR) which play important roles in osteogenesis [31, 65]. Moreover, it was also reported that Ta could inhibit the expression of PI3K, an important component of mammalian target of replication (mTOR) signaling pathway, to participate in the regulation of bone metabolism [31].

## 3. Summary and Personal Perspective

Porous Ta has been extensively applied as orthopaedic implants due to its excellent biocompatibility, corrosion resistance, and osteogenesis capacity. The focus of this review is to sketch out an osteogenesis panorama of Ta on the basis of current publications. At present, the underlying signaling pathways through which Ta induced osteogenesis were preliminarily discovered, such as Wnt/*β*-catenin, BMP, TGF-*β*, and integrin signaling pathways. The integration of these mechanisms would provide a novel insight into the study and application of Ta as orthopedic implants. To our best knowledge, this is the first review summarizing the mechanisms of Ta in osteogenesis.

It is well-known that the biological performance of orthopedic implants mainly depends on the inherent properties and surface topography (structures and morphologies) [66, 67]; the different surface topography of the implants may mask the authentically intrinsic osteoinductivity [68]. Therefore, it deserves consideration that studies exploring the underlying mechanisms of Ta in osteogenesis usually employed materials with a highly smooth surface or powders knowingly to eliminate the influence of topography [69, 70].

There were complex cross-talking and interactions throughout different signaling pathways, and the involved pathways regulated each other positively or negatively. Similarly, there was cross-talking in the signaling pathways triggered by Ta. For example, osteogenic differentiation was involved in different signaling pathways activated by Ta, and *β*-catenin activated by Ta not only enhanced the transcription of Runx2 but also induced the expression of Smad6 which was reported to be a negative feedback regulator of BMP signaling pathway [54]. Ta may trigger the regulatory loop of BMPs signaling in bone formation since BMP3 initiated by Ta was able to activate Smad2/3, and this could antagonize the osteogenic capacity of other BMPs by inhibiting the formation of Smad1/5/8 complex [71].

Of course, we must acknowledge that there are some discrepant reports. Chen et al. [72] demonstrated that Ta could influence the biological performance of cells by producing ROS, which was inconsistent with that of Wang et al. [30]. This may be attributed to the methods of manufacturing Ta samples because bioactivity of biomaterial was associated closely with manufacturing methods. The upregulation of BMP2 was observed at 2 weeks after implantation of PTTM, but downregulation was also observed at 4 weeks. This may be because Ta regulated BMP2 expression by special spatial-temporal rule. Some researchers propounded some latent factors accounting for the osteoinductive capacity of Ta. Miyaza et al. reported that the deposition of bone-like mineralization on the Ta surface was due to the presence of the Ta-OH structure [73]. Appropriate hydrophilicity and water contact angle were also regarded as crucial factors for the bioactivities of Ta [74]. It was also reported that the osteogenic property of Ta may result from its distinct elastic modulus [75, 76], because appropriate elastic modulus could conduct the osteogenic differentiation by triggering the integrins [77].

Even though several studies illuminated the underlying osteogenesis mechanisms of Ta applying some classical signaling pathways, some limits are also worth pondering. First of all, subsistent studies explored the signaling pathways triggered by Ta just in preliminary, and some mechanisms were not stated in detail; further in-depth researches for specific mechanisms remain to be done. Secondly, there were no publications investigating whether Ta promotes osteogenesis through other classical pathways associated with bone remolding, such as insulin signaling pathway or NOTCH signaling pathway, because there are complex cross-talking between these signaling pathways [62, 78, 79].

In the future, studies about mechanisms of Ta on osteogenesis should be more comprehensive, in detail and in-depth, and the influence of Ta on osteolysis deserves attention. It is of great significance to investigate the transducing from the mechanical signal of Ta to biological signal, which has been a hot topic in the field of biomaterials currently [76, 80, 81]. There is still a long way to go to explore the mechanisms clearly.

## 4. Conclusion

In recent years, our understanding of the osteogenic action of Ta has remarkably advanced. Current evidence from published studies revealed that Ta modulates osteogenesis through regulation of the Wnt/*β*-catenin signaling pathway, BMP signaling pathway, TGF-*β* signaling pathway, and integrin signaling pathway. These studies give essential explanations to the phenomenon that Ta induces osteogenesis.

## Figures and Tables

**Table 1:** Summary of critical molecules and signaling pathway activated by Ta in osteogenesis.

Studies	Targeted molecules	Signaling pathways	Biological effect
Wauthle et al. [11], Kaivosoja et al. [25], Lu et al. [26], Hefni et al. [27]	BMP2, BMP3, BMP4, BMP5, BMP7	BMP/Smad/Runx	Promote osteogenic differentiation and osteogenesis
Shi et al. [16], Hefni et al. [27]	TGF-*β*2, TGF-*β*3	TGF-*β*/Smad/Runx	Promote osteogenic differentiation
Sollazzo et al. [28], Shi et al. [16]	WISP3, *β*-catenin	Wnt/*β*-catenin	Promote osteogenesis
Lu et al. [22], Zhu et al. [29]	Integrin *α*5/*β*1, FAK	Integrin/ERK1/2	Promote osteogenic differentiation and osteogenesis
Wang et al. [30], Sollazzo et al. [28]	ROS, MAP3K2	MAPK	Promote osteogenesis
Wu et al. [17], Shi et al. [16]	OPG, RANKL	OPG/RANKL.RANK	Promote osteogenesis and inhibit osteoclastogenesis
Stiehler et al. [31]	ATM, ATR	p53 signaling pathway	Promote osteogenic differentiation
Kang et al. [32]	LC3B, P62	Autophagy	Promote proliferation
